# Supplementary material for: Impact of restriction of over-the-counter sales of antimicrobials on antimicrobial resistance in Escherichia coli from community-onset urinary tract infections in inner São Paulo State, Brazil
Source: PLoS One. 2021 Nov 10;16(11):e0259632. doi: 10.1371/journal.pone.0259632 (PMC8580237; doi:10.1371/journal.pone.0259632)
Supplement: Attachment — Submitted filename: RESPONSE TO REVIEWERS.docx [file pone.0259632.s002.docx]

To the Editor –

We thank you and the reviewers for carefully reading our manuscript and for your useful recommendations. We responded each question bellow and changed the manuscript accordingly.

The authors

PONE-D-21-00235

Impact of restriction of over-the-counter sales of antimicrobials on antimicrobial resistance in Escherichia coli from community-onset urinary tract infections in inner São Paulo State, Brazil.

PLOS ONE

EDITOR

Dear Dr. Fortaleza,

Thank you for submitting your manuscript to PLOS ONE. After careful consideration, we feel that it has merit but does not fully meet PLOS ONE’s publication criteria as it currently stands. Therefore, we invite you to submit a revised version of the manuscript that addresses the points raised during the review process. Please proceed to revise the manuscript paying special attention to the comments provided by reviewer number 2. We look forward to your revised version. For Lab, Study and Registered Report Protocols: These article types are not expected to include results but may include pilot data.  If you will need more time than this to complete your revisions, please reply to this message or contact the journal office at plosone@plos.org. When you're ready to submit your revision, log on to https://www.editorialmanager.com/pone/ and select the 'Submissions Needing Revision' folder to locate your manuscript file. Please include the following items when submitting your revised manuscript. A rebuttal letter that responds to each point raised by the academic editor and reviewer(s). You should upload this letter as a separate file labeled 'Response to Reviewers'.A marked-up copy of your manuscript that highlights changes made to the original version. You should upload this as a separate file labeled 'Revised Manuscript with Track Changes'. An unmarked version of your revised paper without tracked changes. You should upload this as a separate file labeled 'Manuscript'. If you would like to make changes to your financial disclosure, please include your updated statement in your cover letter. Guidelines for resubmitting your figure files are available below the reviewer comments at the end of this letter.If applicable, we recommend that you deposit your laboratory protocols in protocols.io to enhance the reproducibility of your results. Protocols.io assigns your protocol its own identifier (DOI) so that it can be cited independently in the future. For instructions see: <http://journals.plos.org/plosone/s/submission-guidelines#loc-laboratory-protocols>. We look forward to receiving your revised manuscript. Kind regards,

Monica Cartelle Gestal, PhD, Academic Editor. PLOS ONE. ournal Requirements: When submitting your revision, we need you to address these additional requirements. 1. Please ensure that your manuscript meets PLOS ONE's style requirements, including those for file naming. The PLOS ONE style templates can be found at

https://journals.plos.org/plosone/s/file?id=wjVg/PLOSOne_formatting_sample_main_body.pdf and

<https://journals.plos.org/plosone/s/file?id=ba62/PLOSOne_formatting_sample_title_authors_affiliations.pdf> 2. In ethics statement in the manuscript and in the online submission form, please provide additional information about the patient records/samples used in your retrospective study. Specifically, please ensure that you have discussed whether all data/samples were fully anonymized before you accessed them and/or whether the IRB or ethics committee waived the requirement for informed consent. If patients provided informed written consent to have data/samples from their medical records used in research, please include this information.3. Your ethics statement should only appear in the Methods section of your manuscript. If your ethics statement is written in any section besides the Methods, please move it to the Methods section and delete it from any other section. Please ensure that your ethics statement is included in your manuscript, as the ethics statement entered into the online submission form will not be publIshed alongside your manuscript. 4. Please include captions for your Supporting Information files at the end of you manuscript, and update any in-text citations to match accordingly. Please see our Supporting Information guidelines for more information: http://journals.plos.org/plosone/s/supporting-information.

AUTHORS´ RESPONSE: We changed our manuscript as required.

REVIEWERS' COMMENTS:

Reviewer's Responses to Questions

Comments to the Author **1. Is the manuscript technically sound, and do the data support the conclusions?** The manuscript must describe a technically sound piece of scientific research with data that supports the conclusions. Experiments must have been conducted rigorously, with appropriate controls, replication, and sample sizes. The conclusions must be drawn appropriately based on the data presented. Reviewer #1: Yes. Reviewer #2: Partly

AUTHORS´ RESPONSE**:** We have changed the conclusions to be supported by the data we presented. The main conclusion is that we have a significant (p<0.001) impact after the intervention in amikacin and cefazolin.

**2. Has the statistical analysis been performed appropriately and rigorously?** Reviewer #1: Yes. Reviewer #2: I Don't Know

 AUTHORS´ RESPONSE**:** Even though methodological choices and statistical analysis have both advantages and limitations, interrupted time series (ITS) analysis is more appropriate that usual “before and after” comparisons, since: (1) Monthly rates are not independent observations, which are assumptions of tests such as Chi-square, Student T test and Mann-Whitney U test, usually applied to “before and after analyses”; (2) ITS addresses both immediate changes and the impact on long term trends.

**3. Have the authors made all data underlying the findings in their manuscript fully available?** The PLOS Data policy requires authors to make all data underlying the findings described in their manuscript fully available without restriction, with rare exception (please refer to the Data Availability Statement in the manuscript PDF file). The data should be provided as part of the manuscript or its supporting information, or deposited to a public repository. For example, in addition to summary statistics, the data points behind means, medians and variance measures should be available. If there are restrictions on publicly sharing data—e.g. participant privacy or use of data from a third party—those must be specified. Reviewer #1: Yes Reviewer #2: Yes

AUTHORS´ RESPONSE**:** As referred by reviewers, the database was uploaded alongside the manuscript.

**4. Is the manuscript presented in an intelligible fashion and written in standard English?** PLOS ONE does not copyedit accepted manuscripts, so the language in submitted articles must be clear, correct, and unambiguous. Any typographical or grammatical errors should be corrected at revision, so please note any specific errors here. Reviewer #1: Yes. Reviewer #2: Yes

AUTHORS´ RESPONSE**:** We thank the reviewers.

**5. Review Comments to the Author.** Please use the space provided to explain your answers to the questions above. You may also include additional comments for the author, including concerns about dual publication, research ethics, or publication ethics. (Please upload your review as an attachment if it exceeds 20,000 characters) Reviewer #1: I found this manuscript very well written, in a clear and concise manner. All the findings are very well presented and the analysis of data is well done.

The findings are mostly negative which is very interesting considering how much emphasis is put on controlling antimicrobial drug use as a way to control resistance. I feel that the manuscript should be accepted without any changes. **Reviewer #2**: This is an important study investigating impact of restriction of OTC sales of antibiotics on antimicrobial resistance (AMR). This study attempts to address the most important endpoint in this type of research, but also the most challenging; Preventing increase of AMR is the main objective of regulating sales of antibiotics, but is affected by many other factors. The authors use an innovative methodology for assessing the impact of policy measures on AMR (Interrupted-time-series). However, the study still requires some work before it meets the standards of PLOS ONE. Some points of concern below.

**General comments.** My main concern about this study is that no data is provided about the total sales of the antibiotics that have been looked at in the study. This makes it challenging/impossible to estimate the relevance of the findings in this study. Other studies have shown that impact of regulating OTC sales of antibiotics varies greatly among countries, regions and types of antibiotics. It is essential to include the total sales volumes of cefazolin, ceftriaxone, ciprofloxacin, TMP-SMX and amikacin for the region of interest in the analysis to be able to interpret the resistance data. A similar study has been done by Mattos et al. (Journal of Global Antimicrobial Resistance 2017). Please comment on what this study adds to the knowledge we have obtained from this study.

AUTHORS´ RESPONSE**:** We thank the reviewers. Unfortunately, we had no access to antimicrobial sales database (which are not currently available from governmental databases.). The present study was based on microbiology results that are representative of overall community-onset urinary tract infection in this area. We acknowledge that the study by Mattos et al, ‘‘Brazil’s resolutions to regulate the sale of antibiotics: Impact on consumption and Escherichia coli resistance rates’’ also analyzed the impact of RDC No. 44/2010 on the rates of antimicrobial resistance. Their results showed that antimicrobial resistance rates increased for almost all classes of antibiotics tested and that antimicrobial resistance did not appear to be influenced by the evaluated resolutions (RDCs 44/2010 and 20/2011). Their study includes data from 2009 to 2015, with intervention instituted in October 2010. From this, we emphasize that our study is important because it includes a larger time interval, since our data is from 2005 to 2018. The pre-intervention period on our study, in comparison to theirs, is extended, which adds knowledge to Mattos et al study. Considering that changes in antimicrobial resistance evolve, we believe this larger period analysis is an important advantage of our study. In addition to that, our study differs from his because ours analyzes the impact of RDCs no. 44/2010 in the state of São Paulo, region of (Botucatu). It is a different region (Campinas) from the one evaluated by the study of Mattos et al. Mattos et al found that antimicrobial resistance does not seem to be affected by RDCs, a similar result to ours, since we have also concluded that this resolution by itself was not sufficient to cause a statistically significant impact. In our conclusion, we emphasize the importance of evaluating some additional geographic data, including the use of antimicrobials in agriculture and the different populational patterns of gut microbiota. Thus, we conclude that analyzing a population residing in another location in the state of São Paulo is also a positive point of our study.In the study by Mattos et. al, the sale of antimicrobials from 2008 to 2012 was evaluated, and the samples analyzed varied from 2009 to 2015. Despite the disparity between the analysis intervals of these two aspects, we consider that there was no prejudice to the final conclusion of the study. At the same time, we understand the usefulness of assessing the impact of the resolution on the sale of antibiotics and included the lack of sales data as a limitatipn of our study.The desired impact on antimicrobial resistance is still uncertain in Brazil because there is a lack of regional studies that analyze the rates of antimicrobial resistance throughout the country, using RDC 44/2010 as an intervention. Brazil is the fifth largest country in the world and is characterized by important social inequality, with very large regional disparities. In this way, our country has areas with very different demographic, social and economic characteristics, and each region of the country, in fact, presents a productive peculiarity. Considering that, we believe only legislation changes on the sale of antibiotics was not sufficient as an isolated method to fight against increase of antimicrobial resistance, which is reinforced by the study by Mattos et al.

While the study appears to be sound, the language sometimes is unclear (for example, words like ‘straightforward’, ‘in opposite direction’ and ‘relevant’ are used incorrectly), making it difficult to follow. The whole paper needs a quick copy edit.

AUTHORS´ RESPONSE: a quick edit was done by the authors.

**Title and abstract.** The title is appropriate for the content of the article. The abstract is concise and accurately summarizes the essential information of the paper although it could be improved if the authors address the following issues:• Abstract: Please include aim of the study in Introduction instead of Methods • Abstract: Please include numbers in results for clarification of the findings. Also, the authors claim that they found an ‘impact’ on cefazolin and amikacin resistance, but this is not necessarily due to reduction of OTC sales and does not correspond with the conclusions drawn in this article.• Abstract: Conclusion section now only exists of a policy recommendation, but no conclusion is provided based on your results.

AUTHORS´ RESPONSE: We agree with recommendations and changed the text accordingly.

**Introduction.** The introduction covers most important issues regarding banning OTC sales of antibiotics. Some points to consider: • Unclear what you mean by ‘which have a human development index’. Please elaborate. • No information is provided on what the actual impact of the new regulation was in the region where this study has been done. • Further clarification is needed on why antimicrobial resistance is an important outcome, why desired impact on AMR is still uncertain and include more references on reduction of OTC sales of antibiotics in Brazil (see also comment above)

AUTHORS´ RESPONSE: We agree with recommendations and changed the text accordingly.

**Methods.** The Methods section is comprehensive and detailed but there are some points that should be clarified.• Please explain what resistant tests have been conducted and how resistance was defined in this study.• Why did the authors choose to analyse these specific antibiotics?

AUTHORS´ RESPONSE: We included the information as requested.

***Results*** *It is unclear (in the first paragraph) what IRR values refer to and, more important, how to interpret these values.*

AUTHORS´ RESPONSE: We expanded the result sections to explain and provide interpretations for those findings.

***Discussion*** *• The first paragraph reads like it is unclear whether antibiotics play a role in antimicrobial resistance at all. Moreover, the content of this paragraph seems better suited for the introduction. • The second paragraph contains the most important text in the discussion. Would suggest to move that up to the first paragraph. • There is a lot of focus on direct impact of banning OTC sales of antibiotics on AMR in the results sections, but this approach has some limitations since AMR resistance rates evolve and will not change from one day to another.* ***Maybe consider mentioning this as a limitation. Conclusion.*** *The main conclusion is very clear and well formulated. However, new information and references are quoted in the second part of the conclusion that have not been described in the discussion.*

AUTHORS´ RESPONSE: We expanded the limitation section and rewrote the second part of the conclusion.

***Figure*** *• In figure 1 for amikacin, it seems that the data is not suitable for an ITS because of the large differences between the resistance measurements. These deviating findings require clarification. • Figure 1: Please consider including units for Resistance at Y-axis*

AUTHORS´ RESPONSE: We understand that the variation limits the ITS analysis of Amikacin. We chose to include it as a limitation in the footnote.

***6. PLOS authors have the option to publish the peer review history of their article (what does this mean?). If published, this will include your full peer review and any attached files.*** *If you choose “no”, your identity will remain anonymous, but your review may still be made public. Do you want your identity to be public for this peer review? For information about this choice, including consent withdrawal, please see our Privacy Policy. Reviewer #1: No Reviewer #2: Yes: Tom G. Jacobs*

AUTHORS´ RESPONSE: We thank once again the reviewers for their recommendations.
