# Supplementary material for: Impact of restriction of over-the-counter sales of antimicrobials on antimicrobial resistance in Escherichia coli from community-onset urinary tract infections in inner São Paulo State, Brazil
Source: PLoS One. 2021 Nov 10;16(11):e0259632. doi: 10.1371/journal.pone.0259632 (PMC8580237; doi:10.1371/journal.pone.0259632)
Supplement: Attachment — Submitted filename: RESPONSE TO REVIEWERS.docx [file pone.0259632.s003.docx]

To the Editor –

We thank you and the reviewers for carefully reading our manuscript and for your useful recommendations. We responded each question bellow and changed the manuscript accordingly.

The authors

PONE-D-21-00235

Impact of restriction of over-the-counter sales of antimicrobials on antimicrobial resistance in Escherichia coli from community-onset urinary tract infections in inner São Paulo State, Brazil.

PLOS ONE

1. REVIEWERS' COMMENTS: If the authors have adequately addressed your comments raised in a previous round of review and you feel that this manuscript is now acceptable for publication, you may indicate that here to bypass the “Comments to the Author” section, enter your conflict of interest statement in the “Confidential to Editor” section, and submit your "Accept" recommendation.

Reviewer #2: All comments have been addressed

Reviewer #3: All comments have been addressed

AUTHORS´ RESPONSE**:** We thank the reviewers

2. REVIEWERS' COMMENTS: Is the manuscript technically sound, and do the data support the conclusions?

The manuscript must describe a technically sound piece of scientific research with data that supports the conclusions. Experiments must have been conducted rigorously, with appropriate controls, replication, and sample sizes. The conclusions must be drawn appropriately based on the data presented.

Reviewer #2: Yes

Reviewer #3: Yes

AUTHORS´ RESPONSE**:** We thank the reviewers

3. REVIEWERS' COMMENTS: Has the statistical analysis been performed appropriately and rigorously?

Reviewer #2: I Don't Know

Reviewer #3: I Don't Know

AUTHORS´ RESPONSE**:** a natural experiment based on ecological data was the chosen design. The statistical analysis for interrupted time series (ITS) was performed in STATA 14 (Statacorp, College Station, TX). Seasonality was investigated by Box-Jenkins models in NCSS 9.

4. REVIEWERS' COMMENTS: Have the authors made all data underlying the findings in their manuscript fully available?

The PLOS Data policy requires authors to make all data underlying the findings described in their manuscript fully available without restriction, with rare exception (please refer to the Data Availability Statement in the manuscript PDF file). The data should be provided as part of the manuscript or its supporting information, or deposited to a public repository. For example, in addition to summary statistics, the data points behind means, medians and variance measures should be available. If there are restrictions on publicly sharing data—e.g. participant privacy or use of data from a third party—those must be specified.

Reviewer #2: Yes

Reviewer #3: Yes

AUTHORS´ RESPONSE**:** We thank the reviewers

5. REVIEWERS' COMMENTS: Is the manuscript presented in an intelligible fashion and written in standard English?

PLOS ONE does not copyedit accepted manuscripts, so the language in submitted articles must be clear, correct, and unambiguous. Any typographical or grammatical errors should be corrected at revision, so please note any specific errors here.

Reviewer #2: Yes

Reviewer #3: No

AUTHORS´ RESPONSE**:** the authors have reviewed all the text and corrected some typo and grammatical errors.

6. REVIEWERS' COMMENTS: Review Comments to the Author

Please use the space provided to explain your answers to the questions above. You may also include additional comments for the author, including concerns about dual publication, research ethics, or publication ethics. (Please upload your review as an attachment if it exceeds 20,000 characters)

Reviewer #2: I am happy with the adjustments made by the authors and their responses to the questions. I support publication of this paper in PLOS ONE.
Although, in general, the level of scientific English in the manuscript is appropriate, there are still a few typos and grammatical errors.

AUTHORS´ RESPONSE: We thank the reviewer

Reviewer #3: Dear Authors the following statements are ambiguous:

1. on the conclusion section of the abstract ''At the present study, we found that OTC sales restriction did not generally impact on antimicrobial resistance. '' = it is better rewritten in a more informative way.

AUTHORS´ RESPONSE: we agree with recommendations and changed the manuscript according to suggestion.

2.the last statement of the first paragraph on the background section ''Laws restricting or prohibiting OTC sales of antimicrobials have been issued, but their impact is currently not straightforward. (it is better to paraphrase the phrase -----currently not straightforward)

AUTHORS´ RESPONSE: we agree with recommendations and changed the manuscript according to suggestion.

3.on the first statement of 2nd paragraph of the background section---''The population drivers of antimicrobial resistance are complex''. (it is better to change the phrase population drivers to community drivers)

AUTHORS´ RESPONSE: we agree with recommendations and changed the manuscript according to suggestion.

4.the last statement of the third paragraph of the background section ''Most importantly, the impact on antimicrobial resistance is uncertain in Brazil and in our region. (better to say---the impact on antimicrobial resistance is uncertain in Brazil generally and in our region specifically)

AUTHORS´ RESPONSE: we agree with recommendations and changed the manuscript according to suggestion.

5. the statement on the discussion section ''Disappointing as our findings may be, they are evidently not an argument against
restricting OTC sales of antimicrobials.'' is ambiguous. I advise the authors to make it more clear.

AUTHORS´ RESPONSE: we changed the manuscript according to suggestion. Our study did not found evidence of the impact of OTC sales restriction and antimicrobial resistance, but, we don’t have any arguments against the restriction.
